# Simvastatin Enhances Protection against *Listeria monocytogenes* Infection in Mice by Counteracting *Listeria*-Induced Phagosomal Escape

**DOI:** 10.1371/journal.pone.0075490

**Published:** 2013-09-24

**Authors:** Suraj P. Parihar, Reto Guler, Dirk M. Lang, Harukazu Suzuki, A. David Marais, Frank Brombacher

**Affiliations:** 1 International Centre for Genetic Engineering & Biotechnology (ICGEB), Cape Town Component and Institute of Infectious Diseases and Molecular Medicine (IIDMM), Division of Immunology, Faculty of Health Sciences, University of Cape Town, Cape Town, South Africa; 2 Department of Human Biology, Faculty of Health Sciences, University of Cape Town, Cape Town, South Africa; 3 Division of Genomic Technologies, RIKEN Center for Life Science Technologies, Yokohama, Japan; 4 Division of Chemical Pathology, Faculty of Health Sciences, University of Cape Town, Cape Town, South Africa; Columbia University, United States of America

## Abstract

Statins are well-known cholesterol lowering drugs targeting HMG-CoA-reductase, reducing the risk of coronary disorders and hypercholesterolemia. Statins are also involved in immunomodulation, which might influence the outcome of bacterial infection. Hence, a possible effect of statin treatment on Listeriosis was explored in mice. Statin treatment prior to subsequent *L. monocytogenes* infection strikingly reduced bacterial burden in liver and spleen (up to 100-fold) and reduced histopathological lesions. Statin-treatment in infected macrophages resulted in increased IL-12p40 and TNF-α and up to 4-fold reduced bacterial burden within 6 hours post infection, demonstrating a direct effect of statins on limiting bacterial growth in macrophages. Bacterial uptake was normal investigated in microbeads and GFP-expressing Listeria experiments by confocal microscopy. However, intracellular membrane-bound cholesterol level was decreased, as analyzed by cholesterol-dependent filipin staining and cellular lipid extraction. Mevalonate supplementation restored statin-inhibited cholesterol biosynthesis and reverted bacterial growth in *Listeria monocytogenes* but not in listeriolysin O (LLO)-deficient Listeria. Together, these results suggest that statin pretreatment increases protection against *L. monocytogenes* infection by reducing membrane cholesterol in macrophages and thereby preventing effectivity of the cholesterol-dependent LLO-mediated phagosomal escape of bacteria.

## Introduction

Statins are inhibitors of 3-hydroxy-3-methylglutaryl-coenzyme A reductase (HMG-CoA), a crucial enzyme in cholesterol biosynthesis pathway. Clinically, statins are extensively prescribed drugs to reduce morbidity and mortality in patients with coronary disorders and hypercholesterolemia [1]. Of note, it has also been observed that statin treatment can reduce mortality in patients with active bacteremia [2]. Apart from the ability of statins to decrease mortality in patients with cardiovascular diseases or bacteremia, studies have reported immunomodulatory and inflammatory properties of statins, independent of their ability to lower cholesterol levels [3,4]. A protective role for statins has also been observed in other disease models. Recently, a case study amongst US veterans showed that statin treatment reduced their risk of lung cancer [5]. In addition to cancer, statins have been implicated in controlling Dengue virus replication in human PBMCs [6] and Epstein–Barr virus (EBV)-induced tumor formation in SCID mice was reduced following treatment with simvastatin, resulting in increased survival [7].

Previously, depletion studies using methyl-β-cyclodextrin (MβCD) have shown cholesterol to be crucial for the uptake of various pathogens, including viruses [8], protozoan parasites [9] and bacteria [10,11]. Rapid sequestration of cholesterol by cyclodextrin promotes conformational changes in the membrane and alters lipid rafts, membrane receptors and their intracellular signalling pathways [12,13], which may explain the reduced uptake of pathogens. Statins have also been reported to influence lipid raft-mediated FcγR signalling in human monocytes [14] and lymphocyte signalling by disrupting lipid rafts [15]. Moreover, statins influence neuroprotection by reducing the association of N-methyl-D-aspartate receptors to lipid rafts [16].

The gram-positive bacterium, *Listeria monocytogenes*, which is the causative agent of listeriosis, also exploits cholesterol to invade macrophages. Recent evidence has shown that survival of *L. monocytogenes* in macrophages is dependent on 25-hydroxycholesterol [17]. In order to reside within macrophages, *L. monocytogenes* evades macrophage-mediated killing by expressing their signature virulence factor, known as listeriolysin O (LLO). LLO is a cytolysin which binds with cholesterol to create a membrane pore that allows bacterial escape into the cytoplasmic space for proliferation and dissemination into neighboring cells [18]. It is noteworthy to mention a recent finding on another cholesterol-dependent cytolysin, pneumolysin, which is secreted by *S. pneumoniae*. Here, authors showed that statins have a protective effect on pneumolysin-mediated host cell lysis and bacterial burden in a mouse model of sickle cell disease [19]. This therefore suggests that inhibition of host cholesterol using pharmaceutical agents, such as statins, could potentially influence bacterial escape and alter the disease outcome. While it has been shown that statin treatment can lower bacterial burdens, during *Salmonella enterica* [20] and *Chlamydia pneumoniae* [21] infections in mice, the mechanism behind the antimicrobial activity of statins remains inconclusive. Importantly, statins do not significantly decrease serum cholesterol levels in mice, as rodents express less number of LDL receptors than humans which results in decreased uptake of LDL cholesterol from the blood circulation [22]. More recently, an intriguing finding revealed that statins target the outcome of bacterial infection by forming DNA-based extracellular traps (ETs), an extracellular mechanism responsible for antimicrobial activity in macrophages/neutrophils. This finding indicates that statins can target more than one mechanism *in vivo* [23].

In the present study, we investigated the effect of statin treatment on the growth of the intracellular pathogen, *L. monocytogenes* both *in vivo* and *in vitro*. In summary, we show that simvastatin increases host defense against listeriosis by targeting LLO-dependent escape of *L. monocytogenes*.

## Materials and Methods

### Mice and bacteria

C57BL/6 mice were maintained under specific-pathogen-free conditions within the biomedical animal facility of the Health Sciences Faculty, University of Cape Town. Mice were aged (8-12 weeks) and sex-matched for each experiment. *L. monocytogenes* (EGDe strain) were used for infection [24] and *L. monocytogenes* ∆LLO mutant strain and GFP-expressing *L. monocytogenes* (BUG2377: EGDe-GFP-Cr) was a gift from T. Chakraborty (Institute of Medical Microbiology, University of Giessen, Giessen, Germany) and Edith Gouin (Bacteria cell interactions, Pasteur institute, Paris, France) respectively.

### Ethics statement

All experiments were performed in strict accordance with South African National Guidelines and University of Cape Town of practice for laboratory animal procedures. All mouse experiments were performed according to protocols (Permit number: 012/037) approved by the Animal Ethics Committee of the Faculty of Health Sciences, University of Cape Town. All animal users had successfully completed the mandatory University of Cape Town animal handling courses. All procedures were performed under halothane anesthesia and all efforts were made to minimize suffering.

### Simvastatin treatment and *Listeria monocytogenes* infection in mice

Mice were administered with the indicated doses of simvastatin, pravastatin (Sigma-Aldrich) or phosphate buffered saline (PBS) intraperitoneally daily for one or two weeks as shown in the layout. Following treatment, mice were infected intraperitoneally with *L. monocytogenes* (2x10^5^ CFU) and sacrificed at day 3 post-infection. Bacterial burden and histopathology on lungs and spleens was performed as previously described [24,25].

### Macrophage treatment and infection *in vitro*

Bone marrow-derived macrophages (BMDM) were generated as described [26] and RAW264.7 murine macrophage cell line was a gift from Prof. Gordon Brown, University of Aberdeen, UK. 5x10^5^ cells were cultured in the presence of indicated concentrations of simvastatin or PBS for 24 hours. Cells were then infected with *L. monocytogenes* (MOI=10) or *Listeria* mutant for LLO (ΔLLO) for 1 hour in the presence of simvastatin (50 µM) ± mevalonate (100 µM). At the indicated time points, bacterial growth in cultured cells was determined as previously described [24].

### Cytokine and nitric oxide measurements

Following treatment with simvastatin and IFN-γ (100 Units/ml) overnight, macrophages were infected with *L. monocytogenes* and incubated at 37°C for 1 hour. Cytokines such as IL-12p40, TNF-α and IL-6 in cell culture supernatants were measured by sandwich ELISA whilst nitric oxide (NO) was detected by Griess reagent assay [24].

### Cholesterol content in macrophages and serum

After statin treatment, macrophages were stained for cholesterol using filipin dye as previously reported [27]. Alternatively, cholesterol content was measured in soluble supernatant of total cell lysates using an enzymatic cholesterol assay kit according to manufacturer’s instructions (Bioassay system) [28] or in serum using commercial kit (KAT).

### Cell viability and cytotoxicity

After statin treatment, cellular viability and cytotoxicity was measured by reduction of yellow 3-(4,5-dimethythiazol-2-yl)-2,5-diphenyl tetrazolium salt (MTT) (Sigma-Aldrich) by mitochondrial succinate dehydrogenase enzyme of living cells as previously described [29].

### Phagocytosis assay

After statin treatment, macrophages were incubated with beads (4.5μm, FITC labeled Dynabeads, Invitrogen) at a ratio of 10:1. After 1 hour of incubation at 37°C, non-adherent beads were removed by washing with ice-cold PBS and cells were then fixed in 4% paraformaldehyde [30]. Four random fields were photographed using Carl Zeiss LSM 510 confocal microscope. Quantification of internalized beads was based on more than 100 macrophages per treatment.

### Extracellular growth of *Listeria* in the presence of simvastatin

Tryptic soy broth (TSB) supplemented with simvastatin at the indicated concentrations were inoculated with *L. monocytogenes* and cultured on an orbital shaker for 24 hours on 120 rpm at 37°C. Dilutions of cultures were prepared and plated on tryptic soy agar plates to allow enumeration of bacterial numbers.

### ELISA and Western blot analysis of Listeriolysin O (LLO) expression by *Listeria*

*L. monocytogenes* was diluted (1/10) in TSB containing the indicated concentrations of simvastatin and grown to OD_600_ = 0.6. Culture supernatants were analysed for the detection of LLO by ELISA using rabbit anti-LLO (Abcam) primary antibody and alkaline phosphatase-conjugated goat anti-rabbit (BD Bioscience) secondary antibody. Secreted proteins from culture supernatants were precipitated using 10% ice-cold trichloroacetic acid [31] and Western blotting was performed using horseradish peroxidase-conjugated mouse anti-rabbit (Cell Signalling Technology) as secondary antibody.

### Confocal Microscopy

Simvastatin-treated macrophages were infected with GFP-L*. monocytogenes* for 1 hour. Following washing with warm medium to remove extracellular bacteria, cells were fixed at the indicated time points using 4% paraformaldehyde and subjected to actin staining using Rhodamine Phalloidin (Molecular Probes) for 30 minutes at room temperature. Cells were then mounted in mowiol and images were acquired using Carl Zeiss LSM 510 confocal microscope.

### Statistical analysis

Data is represented as mean values ± SEM. Statistical analysis was performed using the unpaired Student’s *t* test, defining differences to untreated control groups as significant (*, *P* ≤ 0.05; **, *P* ≤ 0.01; ***, *P* ≤ 0.001) unless otherwise stated (Prism software).

## Results

### Host defense against *Listeria monocytogenes* is increased by simvastatin treatment in mice

**Figure 1:**
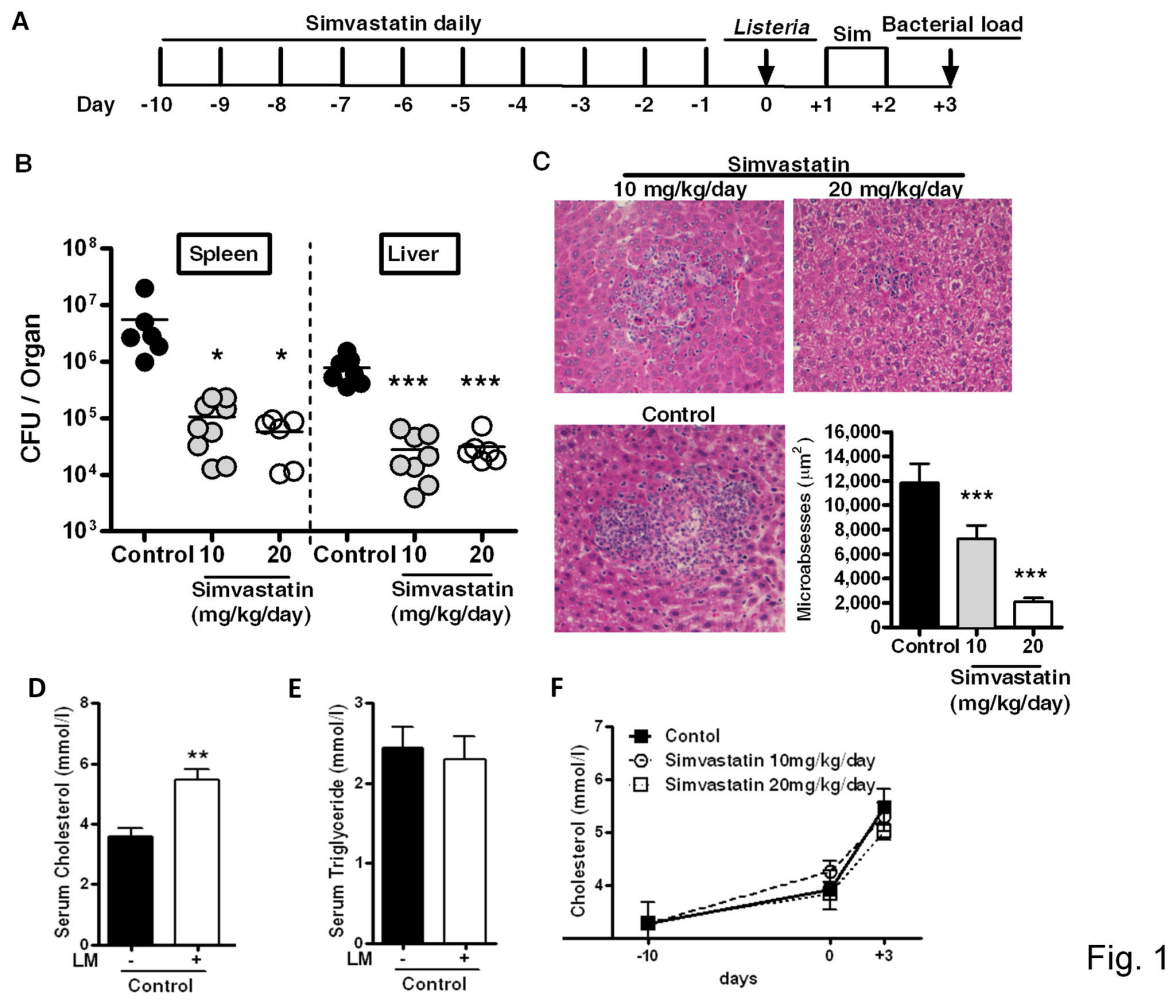
Effect of simvastatin treatment on *Listeria monocytogenes* infection in mice. (A) Mice were treated with simvastatin at 10 and 20 mg/kg/day or with vehicle control by daily intraperitoneal injection for 12 days. Mice were infected intraperitoneally with *L. monocytogenes* (2x10^5^ CFU) and sacrificed as indicated in the layout. (B) Bacterial burden in the spleen and liver determined at 3 days post-infection. Pooled data from two independent experiments are shown. (C) Hematoxylin and eosin stained liver section to determine lesion size in 50-150 microabscesses/group (Original magnification x200). (D) Cholesterol and (E) triglyceride levels were measured in sera before and 3 days after *L. monocytogenes* infection in non-statin treated control mice group. (F) Serum cholesterol levels were measured before and after infection in control and statin-treated groups. Data are representative of two independent experiments. Data are expressed as mean ± SEM of 6-12 mice/group, * *p* < 0.05, ** *p* < 0.01, *** *p* < 0.001 versus control.

We investigated the effect of simvastatin treatment on bacterial burden during the acute phase of *L. monocytogenes* infection in mice. Intraperitoneal administration of 10 and 20 mg/kg/day of simvastatin (Figure 1A) resulted in a 100-fold reduction of bacterial burden in the liver and spleen at day 3 post-infection (Figure 1B). This reduction in bacterial burden was accompanied by well-defined, small hepatic microabscesses in simvastatin-treated mice as revealed by liver histopathology and subsequent quantification of lesion sizes (Figure 1C). Furthermore, *L. monocytogenes* infection significantly increased serum cholesterol (Figure 1D) but not triglycerides (Figure 1E) levels when compared to non-infected mice. In addition, simvastatin treatment alone has no effect on the levels of serum cholesterol in mice (Figure 1F).

**Figure 2:**
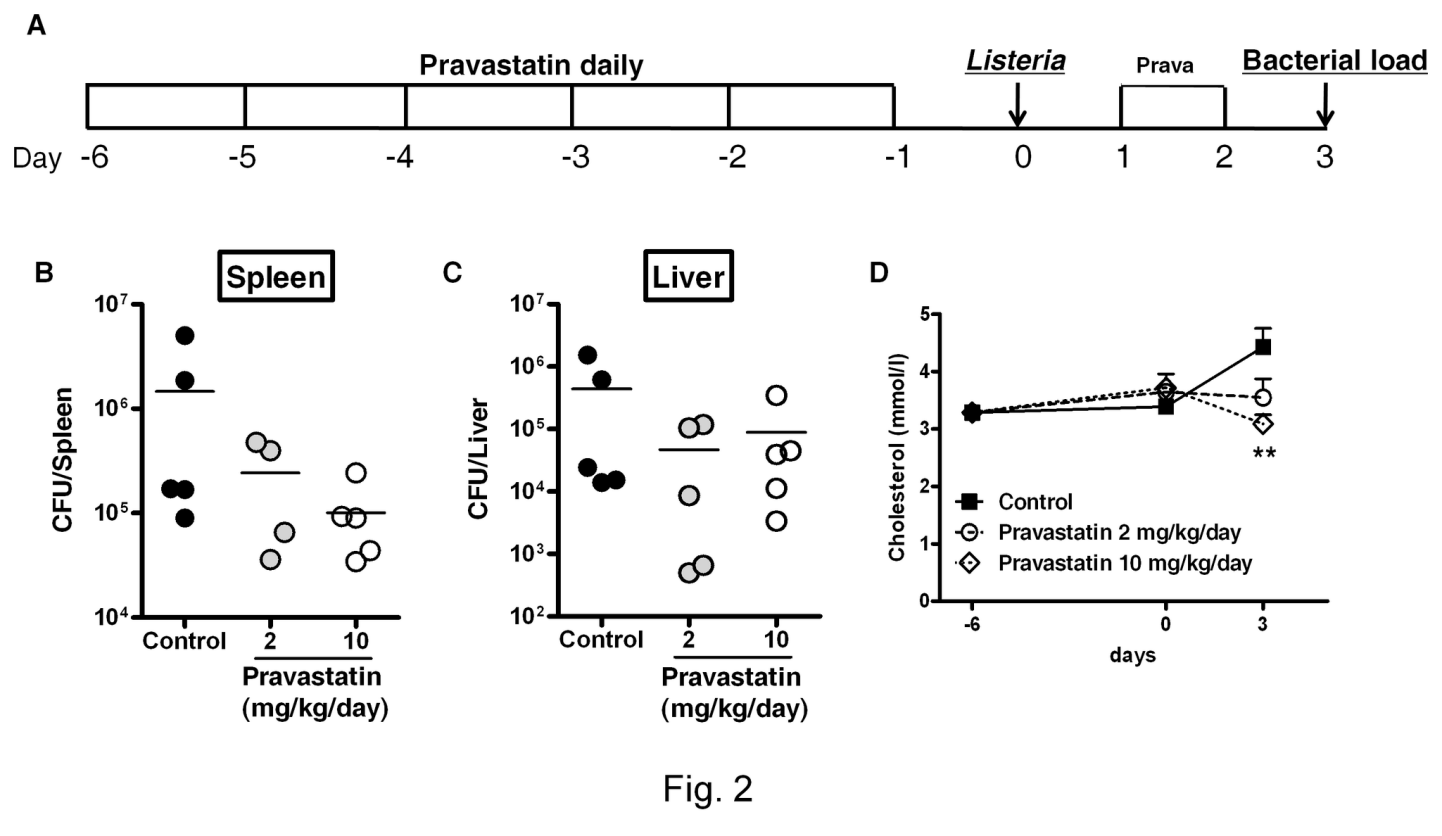
Effect of pravastatin treatment on *L. monocytogenes* infection in mice. (A) Mice were treated with pravastatin at 2 and 10 mg/kg/day or with vehicle control by daily intraperitoneal injection for 8 days. Mice were infected intraperitoneally with *L. monocytogenes* (2x10^5^ CFU) and sacrificed as indicated in the layout. (B) Bacterial burdens in the spleen and (C) liver were determined at 3 days post-infection. (D) Cholesterol levels were measured in sera at indicated times during the course of experiment. Data are expressed as mean ± SEM of 5 mice/group, ** *p* < 0.01 versus control.

We next tested pravastatin, a hydrophilic statin. Mice were treated intraperitoneally with pravastatin at 2 and 10 mg/kg/day as shown in Figure 2A. Pravastatin treatment showed a trend towards decreased bacterial loads in the spleen (Figure 2B) and liver (Figure 2C), when compared to control mice group. At day 3 post-infection, serum cholesterol levels were significantly decreased in pravastatin-treated mice when compared to non-statin treated mice (Figure 2D). The significant reduction in bacterial burden by simvastatin treatment could be attributed to the fact that lipophilic statins can cross the plasma membrane more easily as compared to hydrophilic statins [32]. These results suggest that simvastatin treatment is able to reduce bacterial burden and dissemination to the spleen early during infection with *L. monocytogenes* in mice.

### Simvastatin reduces listerial growth in primary macrophages and a murine macrophage cell line (RAW264.7)

**Figure 3:**
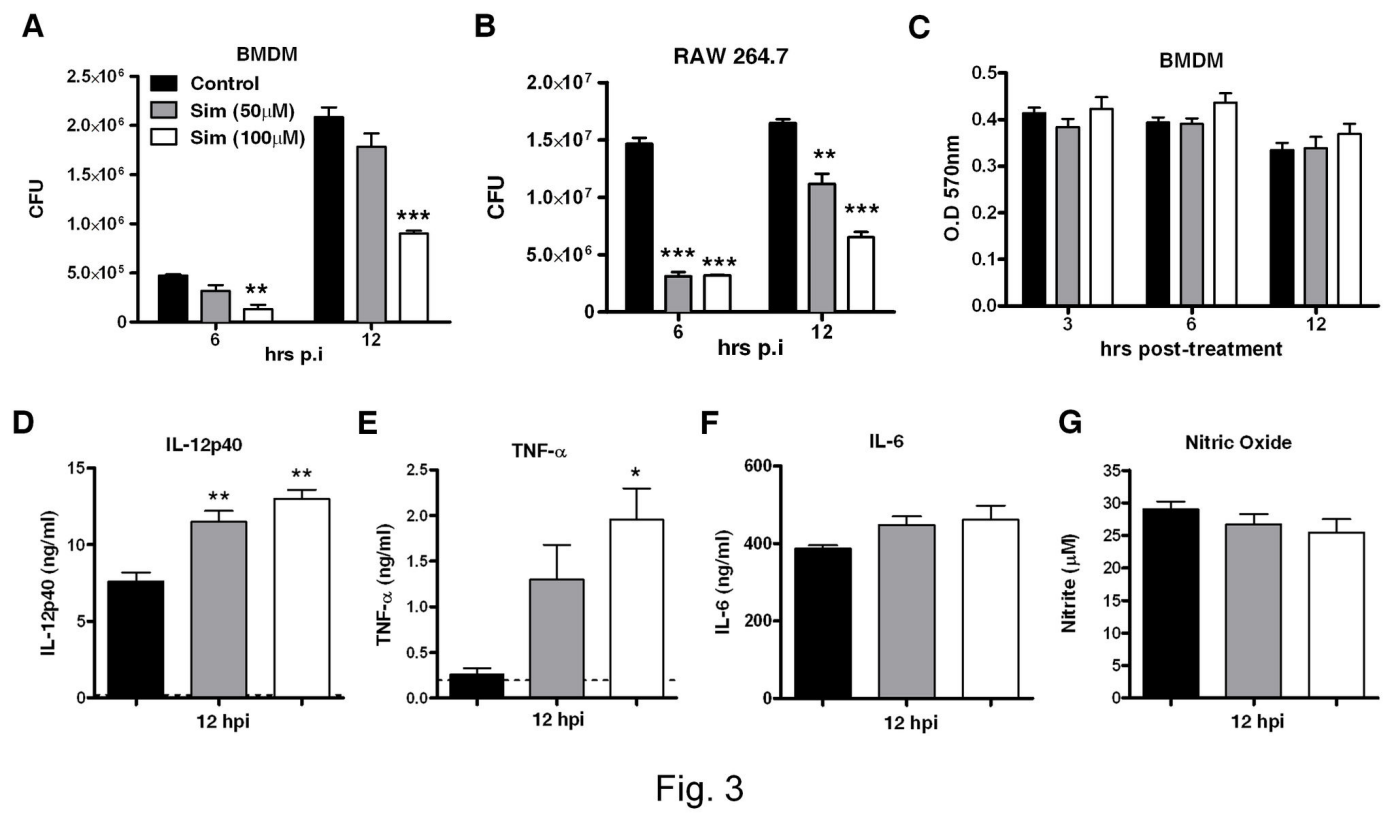
Growth and cytokine profile following *L. monocytogenes* infection in murine macrophages after simvastatin treatment. (A) Murine BMDM and (B) RAW264.7 murine macrophage cell line were pretreated with the indicated concentrations of simvastatin, followed by *L. monocytogenes* infection (MOI=10). Bacterial growth was measured at 6 and 12 hours post-infection. (C) Macrophages were analyzed for statin-mediated cytotoxicity using MTT assay. Following simvastatin treatment and IFN-γ stimulation, macrophages were infected for 12 hours and supernatants were analyzed for the production of (D) IL-12p40, (E) TNF-α, (F) IL-6 and (G) nitric oxide. Results are shown as mean ± SEM of triplicate cultures and are representative of two independent experiments, * *p* < 0.05, ** *p* < 0.01 versus control.

To investigate whether statins could potentially reduce the growth of *Listeria* at a cellular level, primary murine macrophages BMDMs (Figure 3A) and the murine macrophage cell line RAW264.7 (Figure 3B) were treated with different concentrations of simvastatin overnight and subsequently infected with *L. monocytogenes*. At the indicated time points after infection (Figure 3A, B), growth of *L. monocytogenes* was significantly reduced both in BMDMs and RAW264.7 macrophages when treated with 50 µM and above of simvastatin. To rule out the possibility of simvastatin-mediated cytotoxicity in cells, we performed MTT assays to assess the viability of primary macrophages. As shown in Figure 3C, no major differences in viability were observed at any of the simvastatin concentrations used. Similarly, simvastatin had no cytotoxic effect on RAW264.7 macrophages (data not shown). Together, these results show that simvastatin mediates intracellular control of *L. monocytogenes* growth in murine macrophages.

### Increased IL-12p40 and TNF-α production by macrophages following simvastatin treatment

Since IL-12 [33,34] or TNF-α [35] have been shown to reduce bacilli burdens during bacterial infection, We evaluated if statin treatment also enhanced IL-12p40 and TNF- α production in BMDMs. Macrophages were treated with simvastatin and stimulated with IFN-γ for 18 hours and subsequently infected with *L. monocytogenes*. By 12 hours post-infection, simvastatin significantly enhanced the production of IL-12p40 in a dose-dependent manner (Figure 3D), which was accompanied by a significant increase in TNF-α (Figure 3E) whilst non-infected macrophages did not show IL-12p40 and TNF-α production by simvastatin treatment alone (data not shown). Simvastatin also did not affect IL-6 or nitric oxide production in infected BMDMs (Figure 3F, G). Similar results were obtained with RAW264.7 macrophages (data not shown). Altogether, these results suggest that following simvastatin treatment, the increase in production of pro-inflammatory cytokines in macrophages could have contributed to host protection against Listeriosis.

### Simvastatin decreases cholesterol levels and has no effect on phagocytosis in macrophages

**Figure 4:**
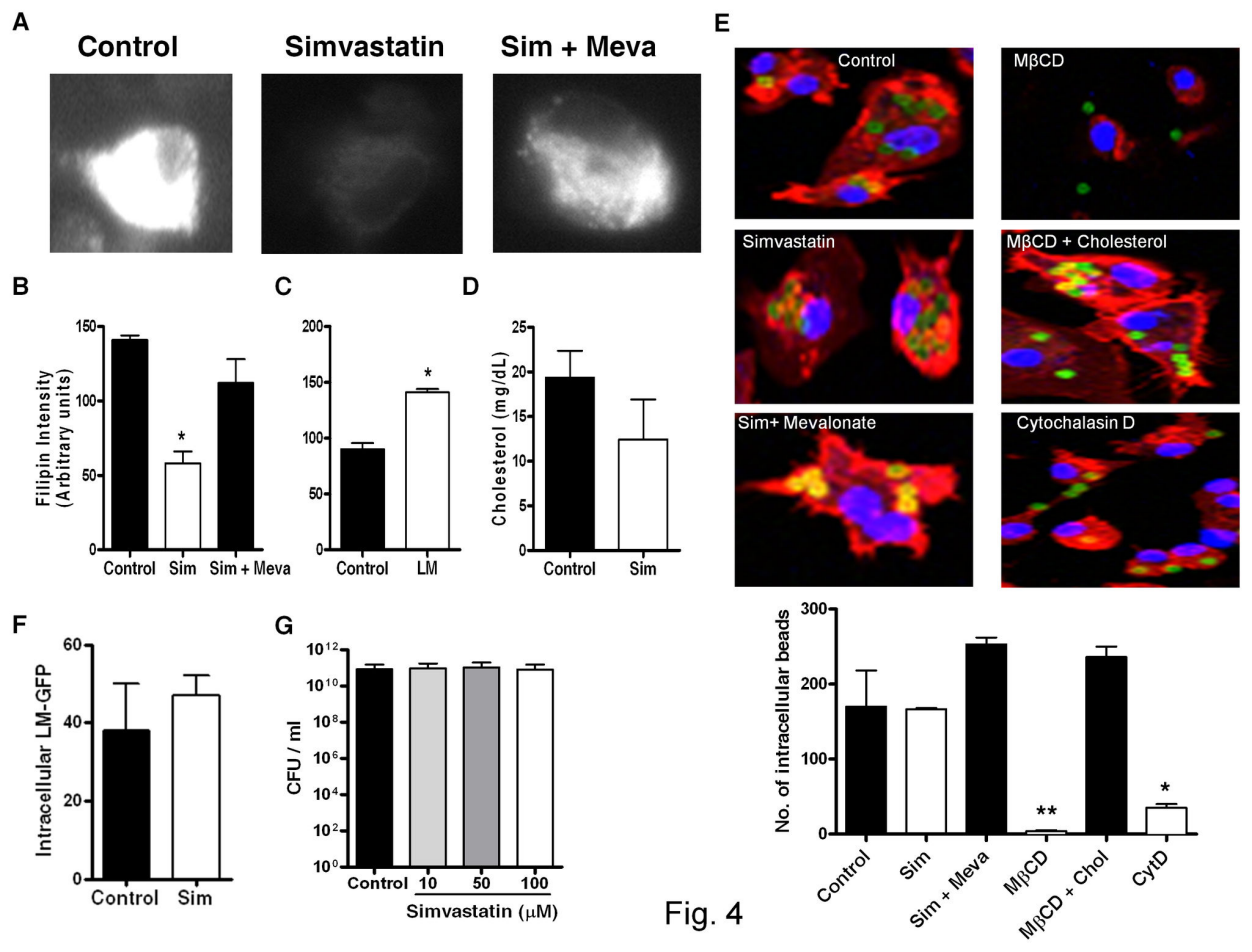
Intracellular cholesterol levels and phagocytosis in macrophages in presence of simvastatin. (A) Representative images of simvastatin ± mevalonate-treated macrophages overnight. Cells were washed and then stained with filipin and (B) fluorescent intensity (arbitrary units) per cell was quantified by Laser Scanning Microscope (LSM) software. Data is shown as intensity from 50-100 cells/group (Original magnification x100). (C) Macrophage cholesterol levels were measured using filipin staining 1 hour after *L. monocytogenes* infection. (D) Cholesterol content was measured in simvastatin-treated macrophage cell lysates following lipid extraction. (E) Macrophages were treated with either simvastatin ± mevalonate for 24 hours or ± methyl-β-cyclodextrin (MβCD) or with cholesterol for 2 hours, and then incubated with latex beads at MOI=10 to measure phagocytosis (Original magnification x100). Cells were then analyzed for number of internalized beads in each setting. (F) Uptake of GFP-expressing Listeria was measured in simvastatin-treated macrophages at 90 minutes post-infection. (G) Extracellular growth of *L. monocytogenes* was determined in tryptic soy broth supplemented with indicated concentrations of simvastatin. Results are shown as mean ± SEM of triplicates and are representative of two or three independent experiments, * *p* < 0.05, ** *p* < 0.01 versus control.

To test whether simvastatin decreases membrane host cholesterol biosynthesis, macrophages were stained with filipin, a fluorescent dye that specifically binds to cell membrane cholesterol. Treatment of macrophages with simvastatin significantly decreased filipin intensity whilst, supplementation of mevalonate restored filipin intensity (Figure 4A, B). In line with our previous observations *in vivo* (Figure 1D), cholesterol levels in macrophages were significantly increased following *L. monocytogenes* infection (Figure 4C). Next, we used lipid extraction to directly quantify the content of intracellular cholesterol in macrophage cell lysates. As observed from the filipin-staining assay, total cholesterol content in simvastatin-treated macrophages was reduced (Figure 4D). Together, these results demonstrate that simvastatin is able to decrease both membrane-bound and intracellular cholesterol in macrophages.

The reduced levels of membrane-bound cholesterol may have had an influence on phagocytic uptake, which would explain the decreased *L. monocytogenes* bacterial growth observed in macrophages. This hypothesis was tested by macrophage internalization studies with latex beads using confocal microscopy. Internalization of beads within one hour of incubation was similar in control and simvastatin-treated macrophages. In addition, we observed reduced uptake of beads in macrophages-treated with methyl-β-cyclodextrin (MβCD), which was reversed upon treatment in combination with cholesterol (Figure 4E). Cytochalasin D, a potent inhibitor of phagocytosis, served as positive control and inhibited phagocytosis in macrophages (Figure 4E). Furthermore, uptake of GFP-expressing *L. monocytogenes* was not impaired by simvastatin treatment (Figure 4F). Taken together, these results suggest that simvastatin has no effect on the phagocytic capacity of macrophages.

### Simvastatin has no effect on extracellular growth of *L. monocytogenes* in culture broth medium

We next investigated whether simvastatin has a direct bactericidal effect on the growth of *L. monocytogenes* in culture medium. To test this hypothesis, we measured bacterial growth in culture medium containing different concentrations of simvastatin. No differences in bacterial growth were observed between control and simvastatin-supplemented culture broth (Figure 4G), indicating that simvastatin at the concentrations used for this study, has no direct effect on the HMG-CoA reductase of *L. monocytogenes*. This result suggests that the reduced bacterial growth of Listeria observed in macrophages was not due to a bactericidal effect of simvastatin directly on *L. monocytogenes*.

### Simvastatin decreases listerial growth in macrophages by interfering with LLO-dependent escape of *L. monocytogenes*

**Figure 5:**
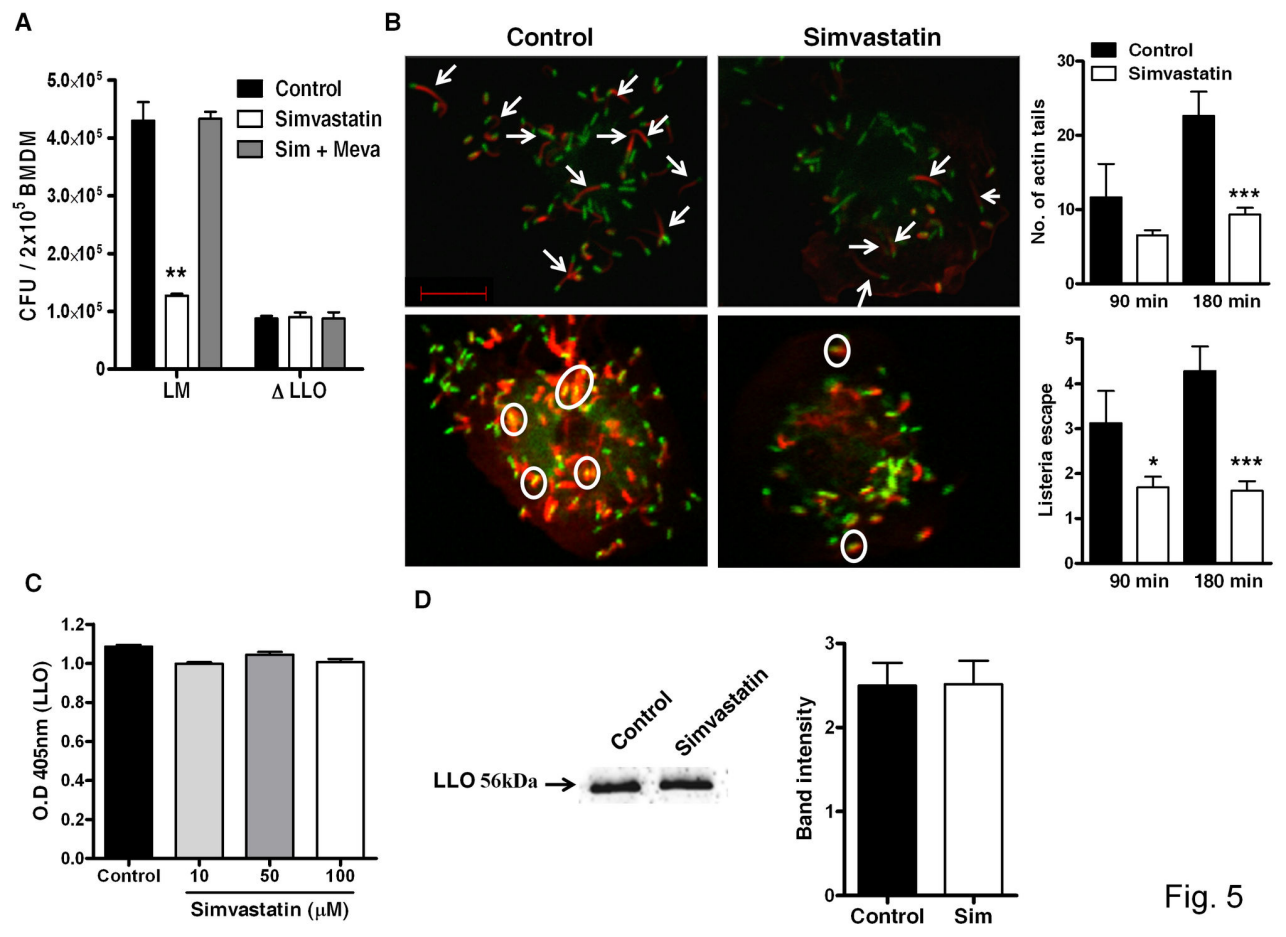
Phagosomal escape of *L. monocytogenes* in macrophages treated with simvastatin. (A) BMDM were treated overnight with simvastatin (100 µM) ± mevalonate (100 µM) and infected with either *L. monocytogenes* or LLO mutant *L. monocytogenes* (MOI=10) for 1 hour. After 12 hours, viable bacilli were determined. (B) Representative images showing actin tails (white arrows) and phagosomal escape (white circles) in simvastatin-treated and control macrophages followed by quantification during the course of Listeria infection (Scale bar = 10µm). Production of LLO secreted by Listeria in presence of indicated concentrations of simvastatin measured by (C) ELISA and confirmed by (D) Western blot analysis. Results are shown as mean ± SEM of triplicate cultures and are representative of two or three independent experiments, * *p* < 0.05, ** *p* < 0.01 and *** *p* < 0.001 versus control.

Furthermore, we determine if the effect on bacterial growth was a consequence of inhibition of the cholesterol biosynthetic pathway, exogenous mevalonate, a precursor in the biosynthetic pathway of cholesterol downstream of HMG-CoA reductase, was added on simvastatin-treated cells in order to restore cholesterol biosynthesis. Supplementation of mevalonate completely abrogated the simvastatin-mediated decrease in bacterial growth (Figure 5A). Listeria secretes a cholesterol-dependent cytolysin, listeriolysin O (LLO), which is crucial for its escape into the cytoplasm [36]. Interestingly, bacterial growth in macrophages infected with a *L. monocytogenes* mutant strain lacking listeriolysin O (∆LLO) was not affected by treatment with either simvastatin or mevalonate (Figure 5A). To directly determine whether simvastatin-mediated impaired Listeria growth was due to the inhibition of bacterial escape from the phagosomes, macrophages were infected with GFP-expressing *L. monocytogenes* (green). Actin staining (red) was then performed using rhodamine phalloidin to identify cytoplasmic bacteria (yellow; merge) and actin-forming tails of cytoplasmic bacteria by confocal microscopy (Figure 5B). Listeria found escaping into the cytoplasm and acquiring actin tails was strikingly reduced in simvastatin-treated macrophages when compared to untreated controls. The possibility that simvastatin treatment might have a direct effect on LLO could be excluded since LLO production by Listeria was not affected by simvastatin treatment, as quantified by ELISA (Figure 5C) and Western blot analysis (Figure 5D) from Listeria culture supernatants. Together, these results suggest that simvastatin provides protection against listeriolysin-mediated cytolysis. Hence, mevalonate-mediated cholesterol biosynthesis plays an important role in listerial growth within macrophages.

## Discussion

In the present study, we report for the first time that statins induce host protective immunity against *L. monocytogenes* infection in mouse model of acute listeriosis. Our results show that simvastatin therapy reduced bacterial burden and subsequent dissemination to primary target organs. This reduction in bacterial titers was accompanied by smaller microabscesses in the livers of statin-treated mice. These results are consistent with previous reports which showed that administration of atorvastatin led to a 2-fold reduction in *Salmonella enterica* bacterial burden [20]. Following internalization by macrophages, recruitment of cholesterol is increased to Salmonella-containing vacuole (SCV) membranes, which together protect the bacilli from phagolysosomal maturation and degradation by inhibiting the recruitment of Rab proteins [37,38]. Similarly, cholesterol is exploited by listerial virulence factor listeriolysin O (LLO), which binds to cholesterol leading to membrane rupture, bacterial escape and subsequent cell to cell spread [18]. We found that the cholesterol level was increased in mice and in macrophages following Listeria infection. This indicates that Listeria is dependent on the cholesterol pathway, which probably increases the sensitivity of these cells to establish infection in host cells. In addition, the lytic activity of LLO in macrophages is dependent on the enzyme γ-interferon-inducible lysosomal thiol reductase (GILT or Ifi30) present in phagosomes [39]. Moreover, the capacity of GILT to activate haemolysin is not only restricted to LLO, but can also activate Streptolysin O, which is secreted by *Streptococcus pyogenes*, another gram-positive bacterium [39]. Our results show a more profound reduction in bacterial burden when compared to the previous study on *Salmonella* [20], which might be due to the different statin used, different bacterial disease studied, the duration of treatment and/or the route of administration.

To understand the possible mechanisms by which statins inhibit *L. monocytogenes* growth, we investigated if statins were able to protect macrophages from a cytolysin, LLO expressed by *L. monocytogenes*. LLO is required for bacterial escape from the vacuole into the cytoplasm to proliferate and disseminate into neighboring cells via actin comets or tails [40]. We therefore infected macrophages with *L. monocytogenes* deficient for LLO (ΔLLO). The growth of ΔLLO was similar in control and simvastatin-treated macrophages, indicating that simvastatin prevent LLO-mediated cytolysis when infected with wild type *L. monocytogenes*, and in the absence of LLO, this protective effect of statin was lost. Furthermore, we showed that simvastatin treatment reduced the number of Listeria escaping into the cytoplasm and formation of actin tails was also reduced. This shows that simvastatin-treated macrophages were protected against the effects of listeriolysin O, a major virulence factor of *L. monocytogenes*. Hence, the ability of Listeria to proliferate and disseminate from the cytoplasm was affected in the presence of simvastatin. These findings suggest that simvastatin treatment in *L. monocytogenes* infected macrophages reduced intracellular cholesterol and the decreased bacterial growth in these cells was contingent on cholesterol-dependent LLO. Statins therefore appear to counteract LLO-dependent escape of bacteria into the cytoplasm. This finding is consistent with a recent report, which showed that simvastatin treatment on endothelial cells protected the host cell lysis by pneumolysin, a cholesterol-dependent cytolysin secreted by *Streptococcus pneumoniae* [19].

Simvastatin treatment also significantly enhanced the secretion of IL-12p40 and TNF-α as *L. monocytogenes* infection progressed in macrophages. A previous report also showed that simvastatin treatment increases production of LPS-induced pro-inflammatory cytokines in peritoneal macrophages. The authors further demonstrated that simvastatin mediated LPS-induced pro-inflammatory cytokine secretion by inhibiting the prenylation pathway, which is known to be important for post-translational modifications. It is therefore possible that upregulation of IL-12p40 following simvastatin treatment might be due to an inhibition of prenylated proteins rather than depletion of cholesterol [41]. Collectively, these results suggest that the simvastatin-mediated reduction of *L. monocytogenes* growth was partially dependent on cytokine production. Previous report have shown that statin treatment decreases MHC-II expression on human endothelial cells and macrophages and has no effect on constitutive expression of MHC II in dendritic cells and B lymphocytes [3]. Similarly, we also observed that simvastatin treatment suppressed MHC-II expression on IFN-γ-activated macrophages and this inhibitory effect was reversed by the addition of exogenous mevalonate. In addition, we found that simvastatin had no effect on constitutive expression of MHC-II (data not shown).

Recent studies have also shown that statins inhibit cell proliferation of various cell types including cancer cells [42,43]. This could be due to cellular efficacy of statins in cell lines that tends to arrest cell growth. In the present study, we observed a negligible effect on cell viability and phagocytic capacity of simvastatin-treated macrophages. Controversially, statins have been reported to either decrease [44,45] or increase phagocytosis [46,47]. However, our results suggest that the statin-mediated reduced bacterial growth was not due to cytotoxicity or impaired phagocytosis. It is noteworthy to mention that simvastatin also had no direct effect on the extracellular growth of *L. monocytogenes* in broth culture. This suggests that administration of simvastatin at the concentrations used in our study was not detrimental to pathogen uptake and host cell proliferation.

Given the nature of statins such as hydrophilic or lipophilic, half-life and potency, the outcome may vary in disease outcome. In the present study, hydrophilic statin (pravastatin) was not able to significantly lower bacterial burdens in mice and appears to be less effective then lipophilic statin (simvastatin). This could be due to three reasons; firstly, hydrophilic statins crosses the cell membrane primarily through selective membrane carriers as opposed to passive diffusion. Secondly, bioavailability of pravastatin is 17% when compared to simvastatin which is 5%, indicating that simvastatin is readily absorbed through the membranes. Thirdly, hydrophilic statins are administered in an active open hydroxy-acid form whereas lipophilic statins are administered in lactone form, which is then converted into open hydroxy-acid form by the cells [32]. These differences in effects of statins might be attributed to differences in their ability to penetrate the cell membranes.

On the basis of primary structure, sequence alignment and sensitivity to statins, HMG-CoA reductase enzymes are classified into Class I and Class II [48]. Eukaryotes utilize class I HMG-CoA reductase enzymes, which can be inhibited by statins at lower concentrations. In contrast, class II reductase enzymes are utilized by prokaryotes and its inhibition requires 1000-fold higher concentrations of statins than compared to class I enzymes [49]. This could explain the inability of simvastatin to induce a bactericidal effect on the growth of *L. monocytogenes*, which indicates that listerial HMG-CoA reductase was not affected at the concentrations used in our study.

In the present study, the maximum dose of simvastatin administered to mice (20 mg/kg) was higher than that administered to humans (0.1-1 mg/kg) [50]. Since equivalent doses in rodents up-regulate the activity of HMG Co-A reductase by 6- to 10-fold, higher doses are required to induce adequate inhibition [51]. The administration of high doses in rodents is warranted since the animals are pharmacodynamically resistant to the pharmacological effect of statins. Statins are known not to significantly lower serum cholesterol levels due to lower levels of low-density lipoproteins in rodents [20]. Similarly, we observed no change in plasma cholesterol levels following simvastatin treatment in mice. A recent report has shown that *L. monocytogenes* upregulates expression levels of 25-hydroxycholesterol which increases the rate of infection in primary macrophages in an IFN-β-dependent manner. This study further demonstrated that 25-hydoxycholesterol controls expression of CD5 ligand, which inhibits activation of caspase-1 thereby promoting survival of *L. monocytogenes* in host cells [17]. In agreement with this, we also observed that serum cholesterol level in mice and macrophage cholesterol content was increased following Listeria infection, which possibly promoted the survival of *L. monocytogenes* in host cells.

## Conclusions

In summary, our findings reveal a beneficial effect of statins treatment during acute phase of listeriosis. Thus, inhibition of the host mevalonate pathway by statins can induce protective immunity against intracellular pathogens by counteracting different pathogen-evasion mechanisms. In a separate study, we have observed a host protective effect of simvastatin in mice and isolated macrophages infected with *Mycobacterium tuberculosis* (manuscript submitted).
